# Modelling the spatial distribution of the nuisance mosquito species *Anopheles plumbeus* (Diptera: Culicidae) in the Netherlands

**DOI:** 10.1186/s13071-015-0865-7

**Published:** 2015-05-01

**Authors:** Adolfo Ibañez-Justicia, Daniela Cianci

**Affiliations:** Centre for Monitoring of Vectors, Food and Consumer Product Safety Authority, Wageningen, The Netherlands; Faculty of Veterinary Medicine, Utrecht University, Utrecht, The Netherlands

**Keywords:** Species distribution modelling, *Anopheles plumbeus*, Mosquito nuisance, Vector-borne diseases, Random forest

## Abstract

**Background:**

Landscape modifications, urbanization or changes of use of rural-agricultural areas can create more favourable conditions for certain mosquito species and therefore indirectly cause nuisance problems for humans. This could potentially result in mosquito-borne disease outbreaks when the nuisance is caused by mosquito species that can transmit pathogens. *Anopheles plumbeus* is a nuisance mosquito species and a potential malaria vector. It is one of the most frequently observed species in the Netherlands. Information on the distribution of this species is essential for risk assessments. The purpose of the study was to investigate the potential spatial distribution of *An. plumbeus* in the Netherlands.

**Methods:**

Random forest models were used to link the occurrence and the abundance of *An. plumbeus* with environmental features and to produce distribution maps in the Netherlands. Mosquito data were collected using a cross-sectional study design in the Netherlands, from April to October 2010–2013. The environmental data were obtained from satellite imagery and weather stations. Statistical measures (accuracy for the occurrence model and mean squared error for the abundance model) were used to evaluate the models performance. The models were externally validated.

**Results:**

The maps show that forested areas (centre of the Netherlands) and the east of the country were predicted as suitable for *An. plumbeus.* In particular high suitability and high abundance was predicted in the south-eastern provinces Limburg and North Brabant. Elevation, precipitation, day and night temperature and vegetation indices were important predictors for calculating the probability of occurrence for *An. plumbeus.* The probability of occurrence, vegetation indices and precipitation were important for predicting its abundance. The AUC value was 0.73 and the error in the validation was 0.29; the mean squared error value was 0.12.

**Conclusions:**

The areas identified by the model as suitable and with high abundance of *An. plumbeus*, are consistent with the areas from which nuisance was reported. Our results can be helpful in the assessment of vector-borne disease risk.

## Background

Mosquitoes (Diptera:Culicidae) are known to be vectors of a large number of pathogens around the globe and are considered as prime candidates for transmitting (re-)emerging vector-borne diseases (VBDs) in Europe [1]. The increased mobility of humans, that has also increased the mobility of livestock and pathogens, as well as environmental modifications and climate changes can contribute to the (re-)emergence of vector-borne diseases [2]. Furthermore, mosquito bites can cause a considerable nuisance for humans and mammals. Severe nuisance can have negative economic consequences (e.g., in tourism, work productivity outdoors, meat and dairy production) [3]. These nuisance situations can eventually lead to autochthonous VBD cases, when in non-endemic areas infectious reservoirs, either humans (travellers, temporary workers) or animals (livestock, migrating animals) come in contact with high density of mosquito vectors.

**Figure 1:**
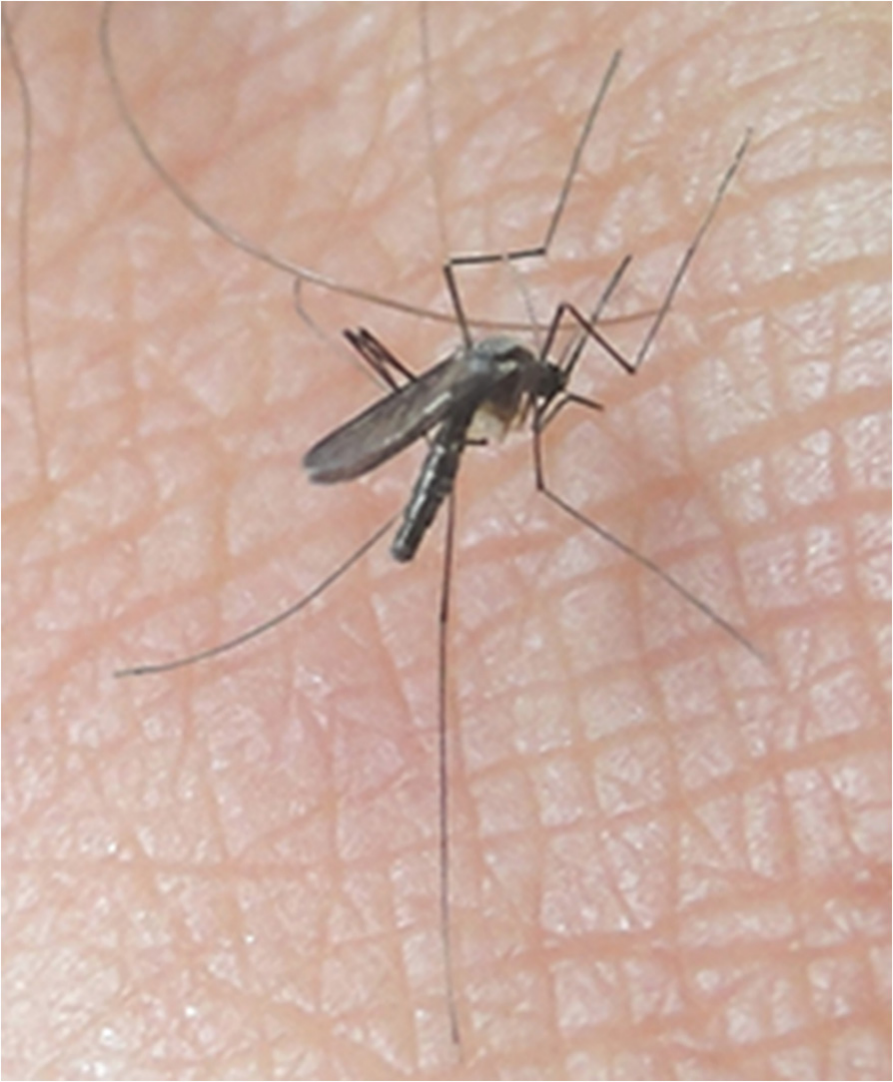
*Anopheles plumbeus* female (source: A. Ibañez-Justicia).

In 2010, in the Netherlands, the Centre for Monitoring of Vectors (CMV) started a nationwide inventory of indigenous mosquitoes to acquire basic information on the composition, geographical distribution, biodiversity and environmental preferences of mosquito species. In this survey, the nuisance mosquito species and potential malaria vector *Anopheles plumbeus* (Stephens, 1828) (Figure 1) was one of the most frequently collected mosquito species [4]. This species has been proven to be able to transmit *Plasmodium falciparum* (Welch, 1897), the causative agent of malaria tropica [5]. Circumstantial evidence for local transmission of *P. falciparum* malaria by *An. plumbeus* has been reported for Germany [6]. *Anopheles plumbeus* has also been incriminated as a vector of *Plasmodium vivax* (Grassi & Feletti, 1890) [7,8] and has been proven to be a laboratory vector of West Nile virus [9]. Even though the health care system is likely to rapidly identify malaria patients and thereby prevent the building up of an infectious human reservoir of *Plasmodium* parasites [10], it is important to gain information on the spatial distribution of *An. plumbeus*, in order to inform the health care system on the areas at risk.

*Anopheles plumbeus* is a mosquito species commonly found in forests, where larvae are usually found in water in rot-holes of trees with high salinity and deficiency of oxygen [11]. They can also be found in containers with stagnant rain water and groundwater, such as tyres, rainwater casks and cemetery vases [5,12]. In the last decade, this species has also been associated with abandoned stables where it breeds in the rain water collected in the manure cellars [13]. This species is known to be a particularly aggressive biter, feeding at any time of the day on different mammalian hosts (including humans), and to a lesser degree on birds and reptiles [14]. In June 2006, nuisance caused by *An. plumbeus* was reported for the first time in the Netherlands, near the city of Nijmegen [1]. Since then, *An. plumbeus* nuisance have been reported every year in the Netherlands, mostly in proximity to abandoned pig stables (Ibanez-Justicia, unpublished).

An understanding of the spatial extent of potential vector species, their abundance and seasonal activity, is important for estimating levels of risks of VBDs and enabling better targeting for surveillance and control. In order to develop basic reproduction number (R0) models and construct risk maps that indicate the risk for an outbreak after an introduction, abundance data of vectors are an essential parameter [15,16]. Although vector presence and abundance are not the only factors determining whether or not a pathogen can spread in an area, determining the distribution of the vector is an essential step in studying the risk of transmission of a pathogen. Given the nuisance and potential risk for the human health, such information on *An. plumbeus* is needed. Currently, no information on the potential spatial distribution of this species is available for the Netherlands.

In this study, we modelled the potential spatial distribution, expressed in occurrence (predicted probability of presence) and abundance of *An. plumbeus* in the Netherlands, based on data collected during the National Mosquito Survey and environmental data. The occurrence was modelled to predict the environmental suitability of the species using a random classification forest model. The abundance was modelled using a random regression forest model with the aim to identify areas where mosquito peaks could be expected. Random (classification and regression) forest models allow external validation through a bootstrapping procedure. The occurrence model was validated also with an external dataset. The resulting maps are in agreement with the reported nuisance for this species and the predictions show a good matching with an external dataset used to validate the model.

## Methods

Species distribution modelling links the occurrence or the abundance of species with environmental data and estimates the similarity of the conditions at any site based on the conditions at the locations of known occurrence/abundance of a species. Here we describe the mosquito data collection, the environmental data used and the statistical methods applied in this study.

### Mosquito data

Mosquito data used for the modelling were obtained from the national mosquitoes survey that was carried out from April to October 2010–2013 by the Dutch Centre for Monitoring of Vectors. Mosquitoes were captured using CO_2_ baited Mosquito Magnet Liberty Plus MM3100 (Woodstream® Co., Lititz, USA). Traps were randomly distributed in the country following a cross-sectional study design, with the following constraint: 40% of the traps were placed in urban areas, 40% in rural-agricultural areas and 20% in natural areas [4]. Urban and agricultural areas were sampled more intensively, because of the potential higher human and veterinary health risk in those areas due to higher exposure.

Data consisted of mosquito abundance data, sampled at 778 locations. For this study the abundance data were reclassified into data of presence (when at least one mosquito was found in the trap) and absence (when no mosquitoes were found in the trap). Each of the locations was sampled only once and each trap was active for one week. The content of the traps was collected weekly and sent to the CMV laboratory. In the laboratory, mosquitoes were morphologically identified using, among others, the Culicidae key specifically designed for rapid field-identification of Dutch adult Culicidae (modified key after Snow [17], Schaffner et al. [9], Verdonschot [18], Becker et al. [19]). Twenty-seven mosquito species were found in the National Mosquito Survey and *An. plumbeus* was the 7th mosquito species most commonly found in the Netherlands. This species was active in the whole period of the survey, from April until October [4]. When a presence and an absence point were in the same square kilometre only the presence point was used because presences inform about the places that are environmental suitable for a species, but absences do not necessarily indicate the opposite [20]. This reduced the number of locations used in the analysis from 778 to 766.

For the validation of the occurrence model, data on *An. plumbeus* presence from confirmed nuisance notifications and data from other mosquito surveys carried out by the CMV in the Netherlands during the years 2010–2014 were used (Table 1). The mosquito data from these surveys were collected with various trapping methods: dippers, pooters, CDC miniature light traps Model 512 (John W. Hock Company, Gainesville, USA), BG Sentinel traps (Biogents AG, Regensburg, Germany) and Mosquito Magnet traps. These data were extracted from VecBase, a tailor-made application built for CMV in 2010 for vector surveillance data.

**Table 1:** **Surveys used for the validation**

**Survey name**	**Year**	**Sampling strategy**	**Capturing device**	**Total nr locations**
EMS-Used tires	2010-2014	Target longitudinal sampling	Larval sampling, manual aspirator, BG-Sentinel trap	16
EMS-Lelystad	2013	Target sampling	MM-Liberty Plus trap	3
NVS-Limburg	2009	Cross-sectional	MM-Liberty Plus trap	14
NVS-Mosquitoes longitudinal	2011	Target longitudinal sampling	MM-Liberty Plus trap	1
Projects	2011, 2012	Target longitudinal sampling	CDC light trap, manual aspirator	3
Nuisance	2010, 2011, 2013, 2014	Check at locations of reported nuisance	Larval sampling, manual aspirator	6
West-Nile-Virus Wetlands	2010	Target longitudinal sampling	MM-Liberty Plus trap, CDC light trap	2

The predictions obtained with the occurrence model that used National Mosquito Survey data were validate with data from these surveys.

EMS: Exotic Mosquito Survey.

NVS: National Vector Survey.

### Environmental variables

The environmental data included in the analysis as predictor variables are 1 km^2^ resolution satellite images and meteorological data in raster file format, commonly used for mosquito distribution modelling [21]. The images were obtained from the MODIS sensor on NASA’s Terra and Aqua satellites [22,23] for 2000–2012 and subjected to temporal Fourier transformation [24,25] to summarise the images and to produce sets of data that capture characteristics of the annual seasonality: the mean, the annual, bi-annual and tri-annual amplitudes and phases, the maxima, minima and variances of the middle infra-red, day and night-time land surface temperature, the enhanced vegetation index and the normalized difference vegetation index signals [26]. Other environmental data used in this study are precipitation (WorldClim [27] and CMORPH [28] 1950–2000), population density (compiled from the Gridded Population of the World Dataset 2000 [29]), the digital elevation model (MODIS [23] 2012) and land cover (Corine land cover map of 2006 [30]). The Fourier components used are provided in Table 2 and the environmental data in Table 3. For each trap location the pixel values of the environmental variables were extracted.

**Table 2:** **Fourier components from temporal Fourier analysis of an imagery time series**

**Component**	**Description**
A0	Fourier mean for entire time series
MN	Minimum value
MX	Maximum value
A1	Amplitude of annual cycle
A2	Amplitude of bi-annual cycle
A3	Amplitude of tri-annual cycle
VR	Total variance
P1	Phase of annual cycle
P2	Phase of bi-annual cycle
P3	Phase of tri-annual cycle
D1	Proportion of total variance due to annual cycle
D2	Proportion of total variance due to bi-annual cycle
D3	Proportion of total variance due to tri-annual and cycle
DA	Proportion of total variance due to all three cycles

Component is the name used in Vecmap.

**Table 3:** **Environmental predictor variables**

**Source**	**Variables**
MODIS	Middle Infra-red (MIR)
MODIS	Day-time land surface temperature (DLST)
MODIS	Night-time land surface temperature (NLST)
MODIS	Enhanced vegetation index (EVI)
MODIS	Normalised difference vegetation index(NDVI)
CMORPH	Precipitation
WorldClim	Precipitation
MODIS	Digital elevation model (DEM)
Gridded Population of the World	Human population density
European Environment Agency	Corine land cover

### Statistical analysis

#### Occurrence model

Three distribution modelling techniques suitable for occurrence data were applied: non-linear discriminant analysis [25], random classification forest [31] and generalised linear model [32]. For each model, the accuracy was assessed using (i) sensitivity, i.e. the ability of a model to correctly identify known positive sites; (ii) specificity, i.e. the ability of a model to correctly identify known negative sites; (iii) the area under the curve, (AUC) that can be roughly interpreted as the probability that a model will correctly predict positive and negative sites [33]. Of the three techniques, random forest provided the best accuracy and therefore the results of this model are presented.

A random classification forest model consists of an ensemble of trees. To create a reliable model, it is generally considered necessary to have the same number of presence and absence points as input. This is because having a different number will create a bias in the model prediction towards the more prevalent category (presence or absence) [33]. For this reason, a ‘balanced’ subset of the data, i.e., a dataset with the same number of presences and absences, was selected. The output produced by the model is an environmental suitability indicator, expressed as a value between 0 (low suitability) and 1 (high suitability). The predictions are visualised in a map with colours ranging from red (high suitability) to blue (low suitability). A list of the most important variables used in the model is given based on the mean decrease in Gini index [31,34]. Random forest allows external validation through a bootstrapping procedure: for each tree, a random subset of the full dataset is sampled with replacement. The model validation is carried out for each tree using the points not used from the full dataset. This validation method is referred to as external, because the model is validated using data that are not used to build the tree. The comparison of the observed and predicted results enables us to calculate accuracy statistics, such as sensitivity and specificity. These measures are calculated for each tree and then averaged to give the overall values.

The predictions produced by the random classification forest were also externally validated against 45 observations from other surveys (Table 1) that reported only the presence of *An. plumbeus.* Comparing the observations obtained with the other surveys and the predictions made by the model using National Mosquito Survey data, the error rate was calculated as the proportion of incorrectly predicted pixels to the total number of points used in the validation.

#### Abundance model

The abundance of the species was modelled using a random regression forest model. The abundance data were transformed according to the formula *log*_*10*_*(abundance + 1)* [35]*.* Because the aim was to identify areas where mosquito peaks could be expected, only the data collected in months in which peaks were observed were selected (June-September). The predicted environmental suitability obtained with the occurrence model described above, was included as one of the predictor variables for modelling the abundance of the species, as it is frequently done in this type of analysis [36-40]. The predicted abundance is interpreted as the expected maximum number of mosquitoes caught in a trap in a certain pixel. The predictions are visualised in a map with colours ranging from light green (low abundance) to dark green (high abundance). The importance of the predictors was assessed using the Increase in Node Purity (INP). The difference between observed and predicted values was expressed as the mean squared error. The analysis has been performed with the software Vecmap demo version [41]. The maps have been produced with Quantum GIS [42].

## Results and discussion

The probability of occurrence (environmental suitability) and the abundance of *An. plumbeus* have been predicted using mosquito field data and environmental data. The estimated environmental suitability and abundance are shown in maps. The important environmental variables used in the models and the accuracy of the models are discussed. The fact that out of three different modelling techniques for occurrence data random forest model was selected based upon its higher classification accuracy is consistent with earlier findings; random forest has been reported to outperform other traditional modelling techniques [43-45].

**Figure 2:**
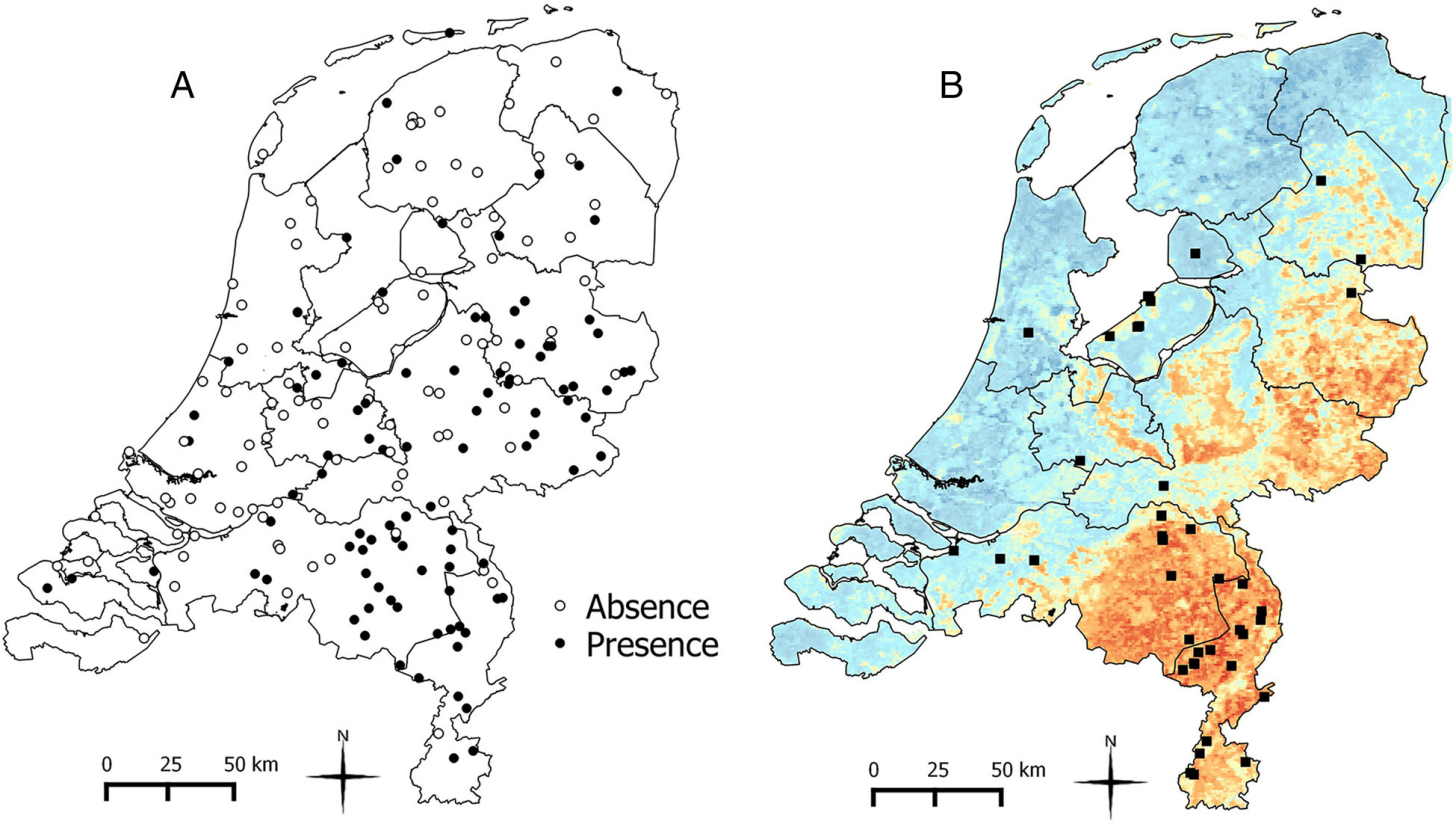
Observed presence and absence points and map of the estimated environmental suitability for *An. plumbeus*. **A**- Presence and absence observed during the National Mosquito Survey program carried out from April to October 2010–2013. **B**- Environmental suitability map of *An. plumbeus* produced using classification random forest. Environmental suitability is depicted using a gradient fill: blue indicates low environmental suitability, red indicates high suitability. Locations where other surveys took place are also shown on the map (black squares).

**Figure 3:**
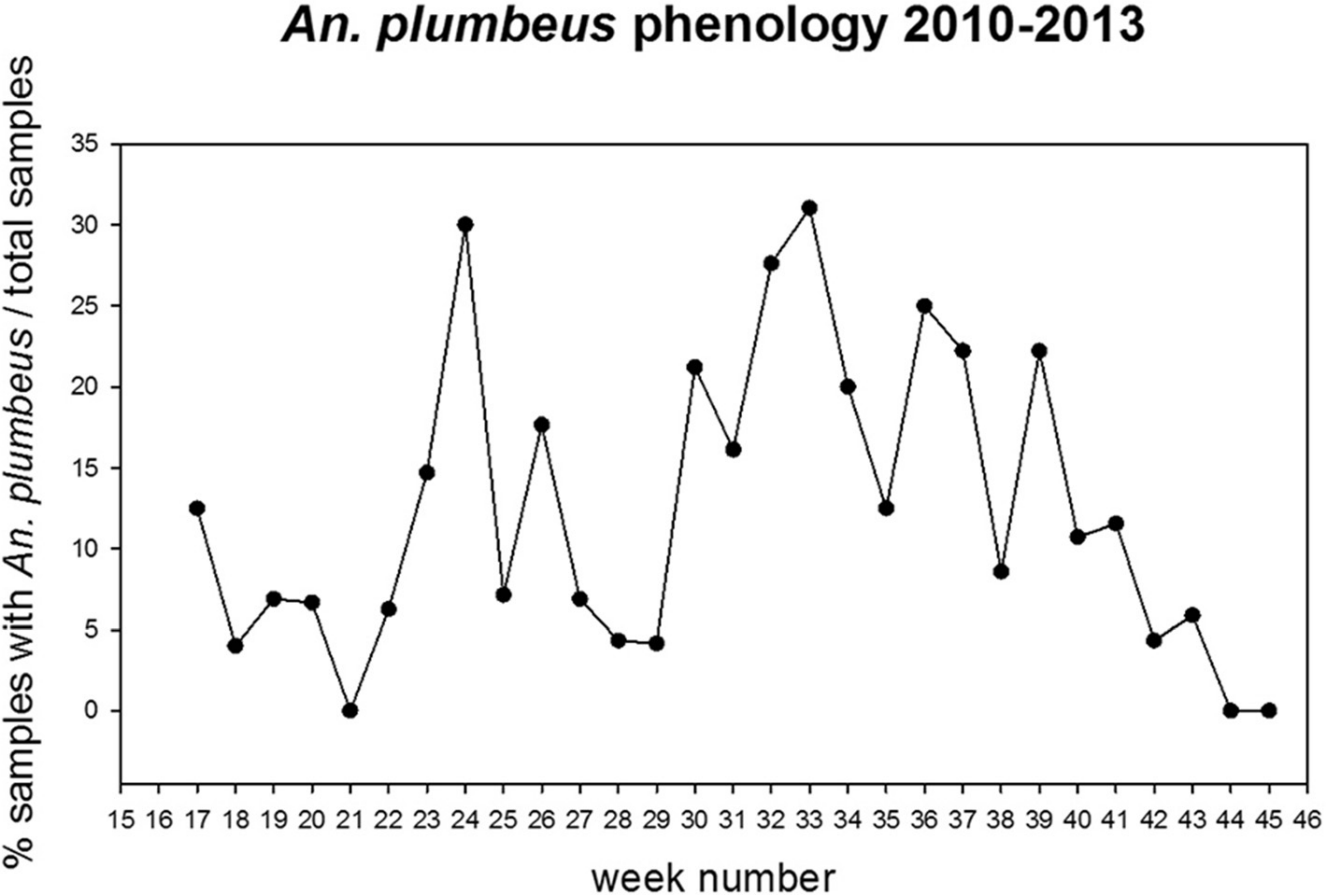
Percentage of positive sites of *An. plumbeus* per week in 2010–2013.

*An. plumbeus* was found in 100 locations out of 766, and it was observed in particular in the eastern part of the Netherlands (Figure 2a). The percentage of traps containing *An. plumbeus* per week is shown in Figure 3. For the modelling, 97 presence points and 97 absence points were selected. Using the random forest model, forest-rich areas in the centre of the Netherlands (e.g. National Park Hoge Veluwe and National Park Utrechtse Heuvelrug) are predicted as suitable for *An. plumbeus* (Figure 2b). Also the eastern parts of the country and in particular the southeastern provinces (Limburg and North Brabant) are predicted to be suitable. In these two provinces nuisance is often reported, especially close to abandoned and un-cleaned pig stables, where mosquitoes breed in manure pools [13]. Based on the environmental characteristics included in the analysis, the model was capable of identifying areas where *An. plumbeus* is truly present, meaning that these characteristics can be a good proxy for abandoned and un-cleaned stables.

Elevation, precipitation, day and night temperature, vegetation indices and middle infra-red (index sensitive to changes in the vegetation) were found to be important predictors for environmental suitability for *An. plumbeus* (Table 4). Precipitation and vegetation are likely to be biologically relevant, since this species typically breeds in water-filled tree holes with high organic material content [19]. Presence of tree-holes is related to the age of the tree or to the tree species. Eggs of this mosquito species are not laid on the water surface but on the sides of potential breeding sites, just above the waterline, so the number of generations produced each year are often dependent upon hydrological conditions [19]. The occurrence model predicts environmental suitability for *An. plumbeus* in areas where old trees with tree-holes are known to occur (e.g., National Park Hoge Veluwe). Even though the species is considered to be a tree-hole breeding species, results obtained using the random forest occurrence model depict environmental suitability for this species in areas without forests in the Netherlands. The results indicate the potential successful use of unforested environments for *An. plumbeus* populations, and imply a similar trend to that seen in continental Europe and UK, where *An. plumbeus* is shifting habitats from almost exclusively breeding in tree-holes to exploiting a wider array of novel man-made larval breeding sites [4,46]. Day and night temperature were found to be related to environmental suitability also in another study in Belgium [1].

**Table 4:** **List of the top 10 most important variables in the occurrence model**

**Rank**	**Variables**
1	DEM
2	CMORPH precipitation, phase of bi-annual cycle
3	CMORPH precipitation, phase of annual cycle
4	Worldclim precipitation, phase of annual cycle
5	Worldclim precipitation, proportion of total variance due to annual cycle
6	MIR, phase of annual cycle
7	NTLS temperature, minimum value
8	DTLS temperature, amplitude of annual cycle
9	NDVI mean
10	CMORPH precipitation, maximum value

The lowest ranking number indicates the most important variable (e.g., rank = 1 is the most important variable). The ranking is based on the mean decrease in Gini index.

Fair accuracy was obtained with the model (AUC = 0.73), which showed a better ability in identifying suitable environments (sensitivity 0.71) than unsuitable environments (specificity 0.66). The accuracy is improved as compared with a first attempt of predicting the environmental suitability for *An. plumbeus* in the Netherlands, where the environmental suitability was extrapolated from Belgium to the Netherlands (sensitivity = 0.50, specificity = 0.49) [1]. The error rate calculated to compare the predicted values to data of other surveys was low (0.29); 71% of the pixels were correctly predicted, meaning that the model could make good predictions in non-sampled areas. However, this is only a partial validation because it considers only presence points and does not give information about the performance of the model in predicting absence points.

**Figure 4:**
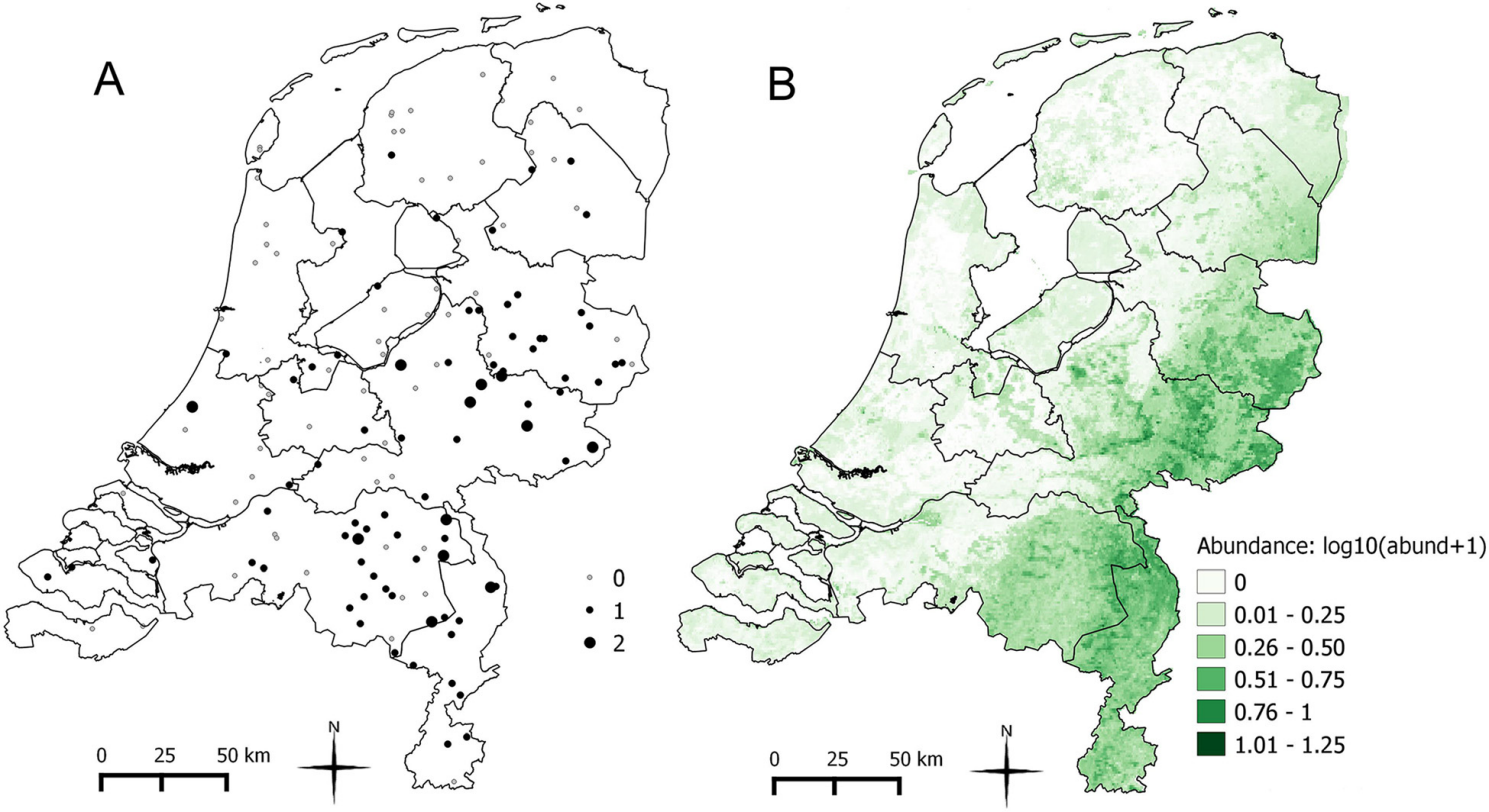
Observed and estimated abundance of *An. plumbeus*. **A** – Observed abundance represented as *log*_*10*_ *(abundance + 1)*. **B** – Map of the estimated abundance produced using a regression random forest. A darker colour indicates higher abundance.

The observed and estimated abundance are shown in Figure 4. In the summer, the observed field abundance was low; rarely more than 10 mosquitoes per trap were found (Table 5). The maximum value observed was 1701 mosquitoes followed by 62 mosquitoes per trap. Given the huge difference between the maximum value and the numbers of mosquitoes observed in the other traps, the maximum value was considered as outlier and excluded from the analysis. However, the reason of this high abundance was investigated and it turned out that the trap was located in a rural area where cattle farms with abandoned stables, not in use anymore, are present. In total, 505 mosquitoes were used in the analysis and they were captured in 80 traps/weeks from 2010 to 2013. The predicted abundance, produced with the random forest technique, was also low (with a maximum of 15 individuals per trap) and the highest abundance was predicted in the eastern part of the country and especially in the south-eastern provinces Limburg and Brabant. These findings are in agreement with the suitable areas identified by the occurrence model. This is not surprising because the probability of occurrence was the most important factor among the environmental factors included in the abundance model (Table 6). Similar results, where the probability of occurrence appears to be the most important factor when using this technique, were already observed in a study conducted on other mosquito and biting midges species in the Netherlands [35]. Other important predictors for abundance of *An. plumbeus* were precipitation and vegetation, in accordance with the biology of the species, as it is explained above. The difference between the observed abundance and the predicted abundance was small, with a mean squared error value of 0.12, meaning that the model predictions matched the observation.

**Table 5:** **Observed abundance used in the model**

**Count**	**Frequency**
0	66
1-10	70
11-20	4
21-30	1
31-40	2
41-50	2
51-60	0
61-70	1

**Table 6:** **List of the top 10 most important variables in the abundance model**

**Rank**	**Variables**
1	Occurrence
2	Worldclim precipitation, phase of annual cycle
3	Worldclim precipitation, proportion of total variance due to bi-annual cycle
4	NDVI, amplitude of annual cycle
5	Worldclim precipitation, amplitude of bi-annual cycle
6	MIR, amplitude of annual cycle
7	DEM
8	NTLS temperature, phase of bi-annual cycle
9	Worldclim precipitation, total variance
10	CMORPH precipitation, phase of bi-annual cycle

The lowest ranking number indicates the most important variable (e.g., rank = 1 is the most important variable). The ranking is based on the Increase in Node Purity (INP).

## Conclusions

The aim of this study was to investigate the potential spatial distribution of *An. plumbeus* in the Netherlands. Using random (classification and regression) forest models, we identified areas with high environmental suitability and high abundance of this species in south-eastern provinces of Limburg and Brabant. These areas coincide with the areas where in recent years most nuisances have been reported. The predictions of the occurrence model were accurate and matched the external dataset used for validation. The abundance model predictions also matched the observation.

The output of species distribution modelling method can be used as an input for risk assessment of establishment and spread of vector-borne diseases [47,48]. Understanding and depicting the potential spatial distribution of mosquito species with modelling techniques is of increasing importance, especially for nuisance mosquito species that can cause economic implications or impact on human health.
